# Fish diversity of Colombian Andes‐Amazon streams at the end of conflict is a reference for conservation before increased land use

**DOI:** 10.1002/ece3.11046

**Published:** 2024-03-13

**Authors:** Juan David Bogota‐Gregory, David G. Jenkins, Astrid Acosta‐Santos, Edwin Agudelo Córdoba

**Affiliations:** ^1^ Aquatic Ecosystems Group Instituto Amazónico de Investigaciones Científicas SINCHI Leticia Colombia; ^2^ Biology Department University of Central Florida Orlando Florida USA

**Keywords:** abundance, altitudinal gradient, Amazon piedmont, beta diversity, effective diversity, land use, multivariate analysis, species richness

## Abstract

Reference conditions are difficult to find in the Anthropocene but essential for effective biodiversity conservation. Aquatic ecosystems in the Andes‐Amazon transition zone of Colombia are now at high risk due to expanded human activities after peace agreements in 2016 ended armed conflict because lands formerly controlled by FARC and other armed groups are now prone to agricultural and urban expansion. Particularly, expanding human land use may reduce fish diversity across the altitudinal gradient, especially in the premontane streams (i.e., <500 m a.s.l.) because lands are more amenable to human use than at greater altitudes. We evaluated fish α‐diversity (measured as species richness, total abundance, and effective species number) and β‐diversity (spatial and temporal) in 12 sites over 8 years bracketing the end of armed conflict. All α‐diversity and β‐diversity analyses were evaluated relative to categorical altitude (< or >500 m) and continuous altitude. Strong differences in fish community structure among sites occurred as a function of altitude. Fish communities exhibit altitudinal biodiversity gradients that are consistent in space and time, and that need to be accounted for conservation and management considerations. Our results provide a reference to identify short‐ and long‐term changes due to impending human land use at a critical moment for the conservation of tropical fish diversity. Similar studies in other areas of the upper Amazon Basin are needed to evaluate effects of subsequent human activities on diversity patterns and our study area to compare to reference conditions reported here.

## Graphical abstract

Aquatic ecosystems in the Andes‐Amazon transition zone of Colombia are now at high risk due to expanded human activities after peace agreements in 2016 ended armed conflict. Fish communities exhibit altitudinal biodiversity gradients that are consistent in space and time, and that need to be accounted for conservation and management considerations. Our results provide a baseline to identify short‐term and long‐term changes due to impending human land use at a critical moment for the conservation of tropical fish diversity.

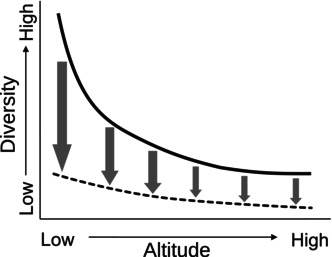

## 1 INTRODUCTION

Increased human activities have already transformed and degraded many ecosystems worldwide due to urbanization, agriculture, and extraction of natural resources (Achiso, 2020; Feng et al., 2022; IPBES, 2019). As a result, diversity is often reduced, measured as a decrease in both species richness and relative abundance (Newbold et al., 2015), and it is difficult to understand reference conditions before human impacts occurred. However, some areas are more affected by humans than others, where differences may be related to human access and landscape suitability for human uses (e.g., agriculture, urban expansion). Among regions undergoing anthropogenic land use change, the Amazon is known for its remarkable biodiversity and endemics (Mittermeier et al., 2003). Amazonian lowlands contain the largest biodiversity in the world (Gentry, 1988; Wilson, 1992), including both terrestrial and aquatic faunas (Myers et al., 2000; Reis et al., 2003). Amazonia hosts about 17% and 10% of all known vascular plants and vertebrate species, respectively (Lundberg et al., 2000; Myers et al., 2000). Freshwater aquatic ecosystems of the Amazon Basin are megadiverse (Myers et al., 2000) and host the most diverse ichthyofauna in the world (Lundberg, 2001; Reis et al., 2003). In highly diverse systems such as the Amazon Basin, many species are relatively rare and occupy specific niches accordingly to their morphological and physiological traits (Hercos et al., 2013). In addition, most species are not evenly distributed, whether measured in presence/absence or in abundance (Bell, 2005; Magurran, 2004; Magurran & Henderson, 2003).

The high freshwater diversity of the Amazon provides crucial ecosystem services, especially fish as food that often represents the sole source of animal protein and financial income for the human population in the region (Agudelo et al., 2006; Agudelo Córdoba et al., 2011). Within Colombia, Amazonian aquatic ecosystems are now at high risk due to expanded human activities, especially expanding agriculture after the establishment of the peace agreements to end armed conflict in 2016 (Tellez, 2019). This scenario sets up a critical moment for the establishment of management and conservation policies (Agudelo Hz et al., 2023; Clerici et al., 2019) to avoid negative conservation outcomes (Feng et al., 2022).

Much of the Amazonian basin is lowland, but not everywhere. The Colombian Amazon includes the Andean‐Amazon transition, where natural ecosystems of basimontane altitudes (i.e., 500–1700 m a.s.l.) are less affected by human activities than those in the premontane zone (i.e., <500 m a.s.l.) because the steep basimontane landscape complicates human activities whereas flat and smooth premontane terrain enables most human activities, despite infertile soils (Galvis et al., 2007).

**FIGURE 1:**
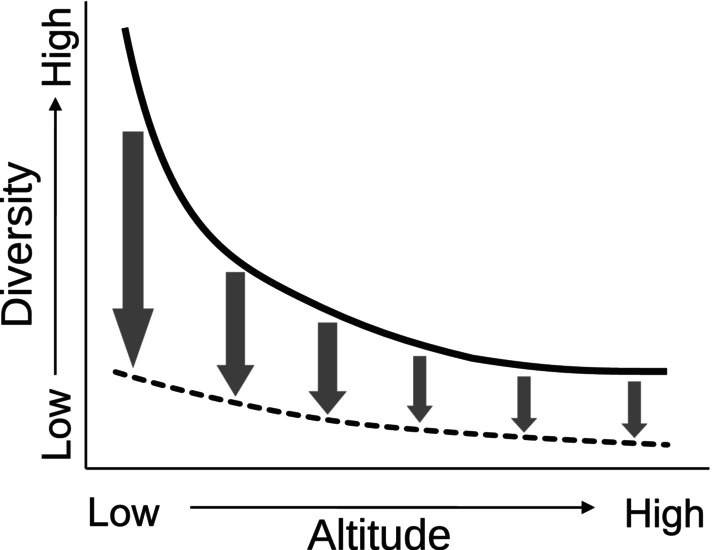
Hypothetical effects of potential land use on fish α‐diversity across elevation gradients. The solid curve represents a decay of diversity with altitude. The lower dashed curve represents potential diversity after natural lands are converted to agriculture and other human uses, where greatest diversity loss (arrows) occurs at lower altitude.

Altitudinal gradients in diversity are well‐recognized worldwide (Keller et al., 2013; Lomolino et al., 2010), where diversity is generally expected to decrease with altitude because more stringent environmental conditions occur at greater altitude (Figure 1; Heegaard & Vandvik, 2009; De La Barra et al., 2016). However, a hump‐shaped diversity ~ altitude pattern is common (Fischer et al., 2011). Environmental conditions along altitudinal gradients are particular to specific locations (Körner, 2007) and the combined effects of these conditions produce diversity patterns (Dyer et al., 2007; Schemske et al., 2009). Different selective conditions across the altitudinal gradient provide different selective conditions, leading to different diversities. For example, basimontane fishes more often have morphological adaptions for attachment to surfaces and behaviors and morphologies to reduce flow forces acting on the fish body (Maldonado‐Ocampo et al., 2005). In comparison, lowland species are more often adapted for less flow forces and instead develop physiological adaptations for warmer temperatures, lower dissolved oxygen, and different pH and conductivity (Bogotá‐Gregory et al., 2020; Saint‐Paul et al., 2000). Therefore, diversity patterns are defined via selection for associated physiological and morphological traits, where the natural conditions act as an environmental filter (Chase & Leibold, 2003). On the other hand, for some taxa, altitudinal patterns might be the result of random mechanisms, which can be described by the mid‐domain effect (Colwell & Hurtt, 1994; Colwell & Lees, 2000).

The Andes‐Amazon transition zone provides a double opportunity to evaluate natural diversity patterns across a strong altitudinal gradient and to evaluate reference conditions for differential and impending anthropogenic effects. The natural altitudinal diversity gradient should interact with spatially biased anthropogenic effects of land use to further modify diversity patterns (Figure 1), where diversity reduction may not be uniform across altitudes (Penjor et al., 2022). Anthropogenic effects may be local and point source (e.g., mining; Rehmana et al., 2024), but more generally extensive, nonpoint source anthropogenic effects occur, related to land use (e.g., agriculture, urban systems). Land use change is often greatest and earliest in lowlands due to simple economics of access and labor (Shively, 2001). Well‐known effects include habitat loss and fragmentation on lands (Adhikari & Hansen, 2018; Plieninger, 2006) and nonpoint source pollution (e.g., sediment loading, nutrient runoff) in streams and rivers (Ikeda et al., 2009; Martinelli et al., 1989). This bias of human activities among terrains seems general, given it is repeated in other landscapes (e.g., Bürgi et al., 2017; Mclain et al., 2013).

Expanding human land use in the Colombian Andes‐Amazon transition zone may reduce diversity but is unlikely to cause biological homogenization between altitudinal levels because the altitudinal gradient is selective (e.g., fishes adapted to low‐slope streams may not move into high‐slope conditions). Instead, premontane streams should become more similar to each other in species richness and abundance, though with different suites of species in lowlands than in uplands (Figure 1). For practical reasons, premontane land use may also be expected to precede that in basimontane regions, with matching timing for diversity effects.

Here we evaluate fish α‐diversity (local species richness, abundance, and effective species number) and β‐diversity (spatial and temporal) patterns in basimontane and premontane streams of the Caquetá River basin in the Colombian Andes‐Amazon transition zone, using data from samples collected between 2013 and 2022 in 12 localities. We expected two general outcomes. First, a greater fish α‐diversity and β‐diversity in premontane streams than in basimontane streams, corresponding to an altitudinal gradient. Second, no great change pre‐ and post‐2016 in patterns because land use had not yet accelerated, because this represents a “transition” period.

Our data represent barely impacted conditions soon after the conflict ended and before the development of formerly avoided lands due to strongholds of FARC and other armed groups (Agudelo Hz et al., 2023; Calle‐Rendón et al., 2018), as well as the COVID‐19 pandemic. Results are especially relevant considering the importance of Andean‐Amazonian connectivity (Anderson et al., 2018; Clerici et al., 2019; Melack & Fosberg, 2001) and the high rates of endemism that characterize the area (Tognelli et al., 2016). Our study sets a baseline for evaluating future changes in Andean‐Amazonian biodiversity while accounting for biodiversity patterns that are fundamental for the elaboration of conservation and management planning. This is particularly relevant in light of environmental degradation, especially under the climate change context.

## 2 METHODS

### 2.1 Study area

The Caquetá River basin is a western Amazonian affluent of Andean origin, formed at 3850 m above sea level (a.s.l.) by the confluence of three different minor tributaries in the Peñas Blancas Páramo, located in the East Mountain Chain in the Southeast Region in Colombia (IGAC, 1996). It runs over 1200 km in a southeast direction before it merges to the main channel of the Amazon River, crossing the Caquetá, Putumayo, and Amazonas departments in Colombia, until it is named the Rio Japurá at the Colombian‐Brazilian border (IGAC, 1996, 1999).

**FIGURE 2:**
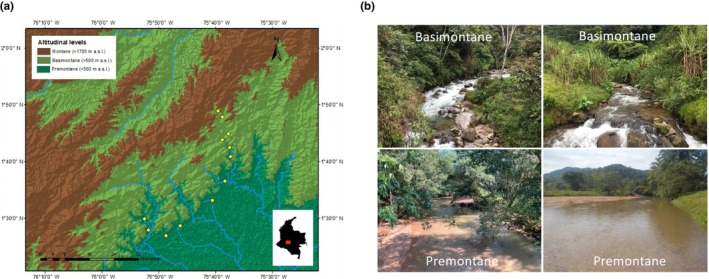
(a) Geographical location of the sample sites. (b) Examples of the aquatic ecosystems within the altitudinal levels, which are the ones recognized by van der Hammen and dos Santos (1995).

The upper section of the Caquetá River drains most of the western uplift of the Guyana Shield, a formation from the Miocene characterized by a crystalline basement. Shields are very evolved soils with low nutrient and organic contents. Above 500 m a.s.l. (Figure 2a), the aquatic systems of the Caquetá́ basin are typically Andean ecosystems with dominant rocky substrates, abrupt slopes, and high flow (Figure 2b). Below the 500 m a.s.l., denoted as Amazonian Piedmont, aquatic ecosystems are characterized by basement alluvial fans (Figure 2b) of volcanic origin with elements from the Andes Mountain chain (Galvis et al., 2007; Hoorn, 1994).

### 2.2 Data collection in situ

The sample sites are located in the Caquetá Department in the municipalities of Belén de los Andaquíes, Florencia, and Morelia, between 200 and 1500 m a.s.l. (Figure 2, Table 1). Sample sites were selected based on location within the basimontane (i.e., >500 m a.s.l.) and premontane levels (i.e., <500 m a.s.l.) and access permission. Sampling occurred during a “transition period” when dialog toward peace agreements was occurring, enabling sites to be accessible. Twelve sites (four sites at the basimontane level and eight at the premontane level) were sampled during the falling water season in each of eight years (2013, 2015–2019, 2021, and 2022). However, three sites were not sampled in 2019 due to access restrictions because of weather conditions. In total, data represent 91 sample events.

**TABLE 1:** Sample localities, geographical coordinates, and altitude (m a.s.l.)

Sites	Locality	Latitude	Longitude	Altitude
S1	La Portada Stream	1.8139	−75.6591	1253
S2	Sucre River	1.7953	−75.6465	1013
S3	Paraíso River	1.7472	−75.6283	676
S4	Las Doradas Stream	1.7289	−75.6464	530
S5	La Sardina Stream	1.6797	−75.6225	386
S6	La Carbona Stream	1.7064	−75.6253	439
S7	La Yuca Stream	1.6078	−75.6383	277
S8	La Mochilero Stream	1.5508	−75.6756	285
S9	Aguas Calientes Stream	1.4758	−75.7694	286
S10	La Chocho Stream	1.4472	−75.8117	316
S11	La Arenosa Stream	1.4644	−75.8633	360
S12	Bodoquerito River	1.4971	−75.8741	382

Fish were sampled using SAMUS725M electrofishing equipment, making multiples passes along a 100 m stretch in each of the sample sites. This sampling methodology follows the standardized sampling technique of Maldonado‐Ocampo et al. (2005) for subsequent data comparisons. Fish were euthanized with clove oil and fixed in a 10% formaldehyde solution. Prior to species taxonomic identifications, specimen vouchers were transferred for preservation in a 75% ethanol solution. All the fish specimens were deposited in the Ichthyological Collection of the Colombian Amazon‐CIACOL at the SINCHI Amazonian Scientific Research Institute in Leticia, Amazonas, Colombia (https://sinchi.org.co/ciacol).

### 2.3 Data analysis

We evaluated the efficiency of our overall sampling effort for basimontane and premontane levels using rarefaction curves and the Chao1 species estimator for data pooled across sample years (Chao, 1984). Extrapolating a species accumulation curve to its asymptote and estimating species richness via rarefaction and the Chao 1, respectively, both provide an estimate of the performance of the proposed sampling method (Chao et al., 2009; Magurran, 2004).

We evaluated diversity components using both univariate and multivariate analyses. We used generalized linear mixed effects models to evaluate the effect of altitude on species richness, total abundance, effective diversity (i.e., e^*H*′^, where *H*′ is the Shannon diversity index; Jost, 2006) and temporal β‐diversity (Jaccard index). Sampling year was a random effect because we used a repeated measures sampling design. Sample sites were also included as a random effect to address unmeasured differences in sites. As a result of this analytical model, results are general for altitudinal effects on fish diversity in the Caquetá River through years, including pre‐ and postconflict time‐periods, and among sites. Alternative models were compared using the corrected Akaike's Information Criterion (AICc) with the bbmle package (Bolker et al., 2022), where we emphasized AICc weights to identify the most plausible model after discounting for model complexity (Burnham & Anderson, 2002). Different alternative residual distributions (e.g., Gaussian, Gamma, negative binomial) in generalized linear models were iteratively evaluated and compared to log‐transforms of response variables to best meet model assumptions (using check_model in the performance package; Lüdecke, 2023). Given a chosen distribution, alternative models were then compared by AICc, where compared models included a null, random effects only, potential fixed effects (i.e., a dummy variable representing pre‐ and postcease fire conditions and altitude effects), and combinations of fixed and random effects. Finally, we used nonmetric multidimensional scaling (NMDS; Bray‐Curtis based) with PERMANOVA to evaluate differences in community structure (β‐diversity). between basimontane and premontane sites.

Data management and statistical analyses were performed in R (R Core Team, 2021) using functions from the glmmTMB (Brooks et al., 2020), vegan (Oksanen et al., 2020), devtools (Hadley et al., 2022), iNEXT (Hsieh et al., 2016), ggplot2 (Kassambara, 2018), MASS (Ripley et al., 2019), multcompView (Graves & Piepho, 2022), and performance (Lüdecke, 2023) packages.

## 3 RESULTS

**FIGURE 3:**
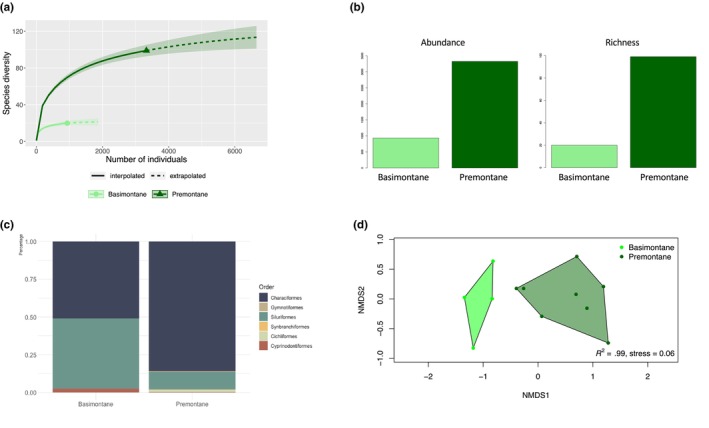
Comparisons of premontane and basimontane fish communities. (a) Rarefaction curves approach asymptotes, indicating representative sampling. Premontane sites were inhabited by more species, as supported by evidence in (b) for total abundance, richness, and effective diversity. (c) Taxonomic composition differed between premontane and basimontane sites, where Siluriformes were more prominent in basimontane sites than in premontane sites. Characiformes were dominant in both site types, but in different proportions. (d) NMDS of fish communities from the sampled sites. Dots represent pooled temporal data (2013–2022) for sites. Polygons represent fish communities based on altitudinal levels recognized by van der Hammen and dos Santos (1995).

Samples included 4216 fish, belonging to 100 species, 58 genera, 24 families, and six orders (Appendix S1). Rarefaction curves reached asymptotes for both basimontane and premontane levels (Figure 3a). Premontane sites had >3 times more fish and >5 times more species than those recorded in basimontane sites (Figure 3b). In addition, basimontane sites had a different taxonomic composition than premontane sites. Basimontane sites had similar numbers of Characiformes and Siluriformes and some (<10%) of the Cyprinodontiformes (Figure 3c). In contrast, the premontane level was clearly dominated (>75%) by the Characiformes fishes which comprised a higher diversity at the order level (Figure 3c). Fish community structure disparities between altitudinal levels are confirmed by the PERMANOVA (*F* = 3.04, *R* = .23, *p* < .05), and the NMDS in which two clear groups, corresponding to the basimontane and premontane sites, are depicted in the multidimensional space representing the analysis (Figure 3d).

**FIGURE 4:**
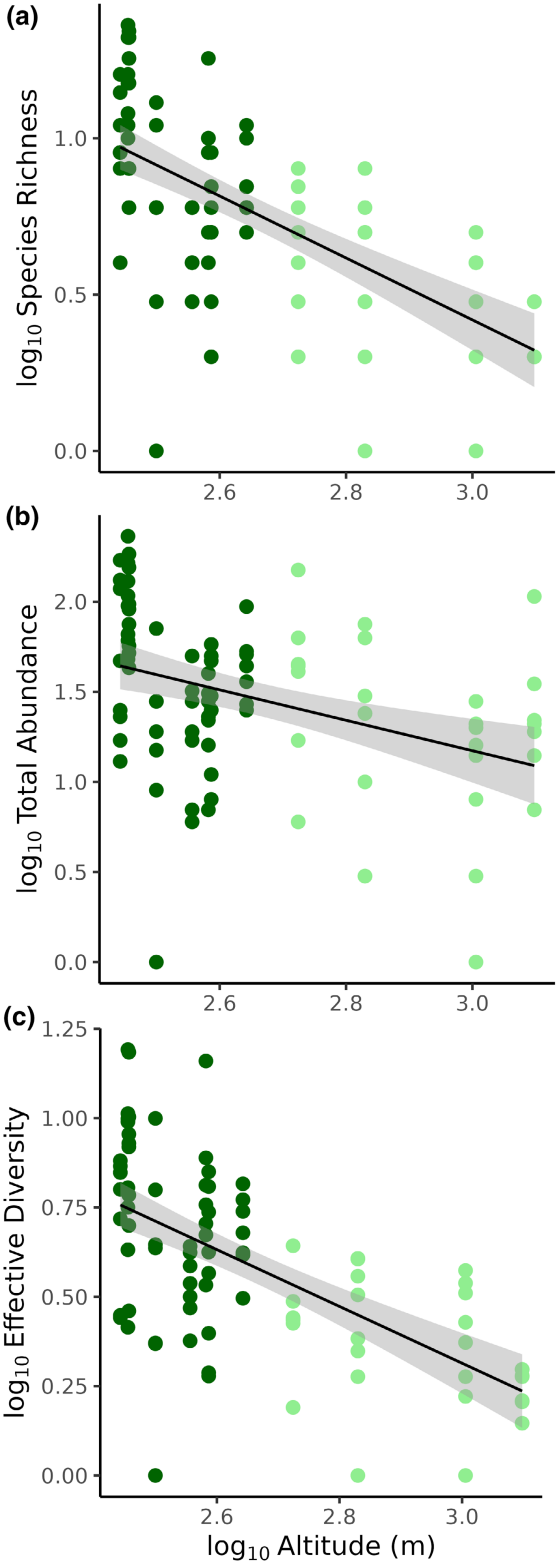
Regression lines for species richness (a), total abundance (b), and effective diversity (c) ~ altitude, where regression lines represent GLMM predicted values and error bars. Note log axes; regressions represent power law functions.

Species richness was most efficiently modeled as a power law (i.e., log–log) function of altitude and the random effects of year and site (Table S1; Table 2). Although altitude alone has a good predictive power (*R* ^2^ = .42) predictions improve with the random effects (*R* ^2^ = .62), as confirmed by the model performance procedure (Figure S1). The model output suggests a significant mean decrease by 0.98 log(species richness) per unit of log‐altitude (Table 2; see Figure 4a for regression line).

**TABLE 2:** Details for the most efficient models to predict α‐diversity species richness, total abundance, and effective diversity (^1^D = exp(Shannon entropy)) of fishes in the Caquetá River, Colombia, based on 91 samples among 12 sites collected in 8 years during the 2013–2022 interval.

	Model distribution	Fixed effects *R* ^2^	Fixed + random *R* ^2^	Random effects	Std. deviation	Fixed effects	Coefficient	95% CI
Species richness	Gaussian	.42	.62	Year	0.1950	Intercept	7.724	2.229
				Sites	0.2612	log(Altitude)	−0.979	0.363
Total abundance	Gamma (log link)	.13	.48	Year	0.359	Intercept	7.987	3.657
				Sites	0.437	log(Altitude)	−0.716	0.594
Effective diversity	Gamma (log link)	.43	.57	Year	0.146	Intercept	6.526	1.622
				Sites	0.170	log(Altitude)	−0.834	1.164

Similar to results for species richness, a power law model for total abundance using altitude as a fixed effect and the random effects was most predictive compared to other models (Table S2; Table 2). Altitude alone represented 42% of variation in species richness, but model predictions improve with random effects (*R* ^2^ = .62; Figure S2). The model output suggests a 0.72 significant decrease in log(fish abundance) per unit of log‐altitude (Table 2; see Figure 4b for regression line).

Effective diversity was also most plausibly modeled as a power law (Table S3; Table 2). Altitude alone has substantial predictive power (*R*‐squared = .433), but adding year as a random effect slightly improved fit (*R*‐squared = .456; Figure S3). The model output suggests a significant 0.834 mean significant decrease in log(effective diversity) per unit of log‐altitude (Table 2; see Figure 4c for regression line).

**FIGURE 5:**
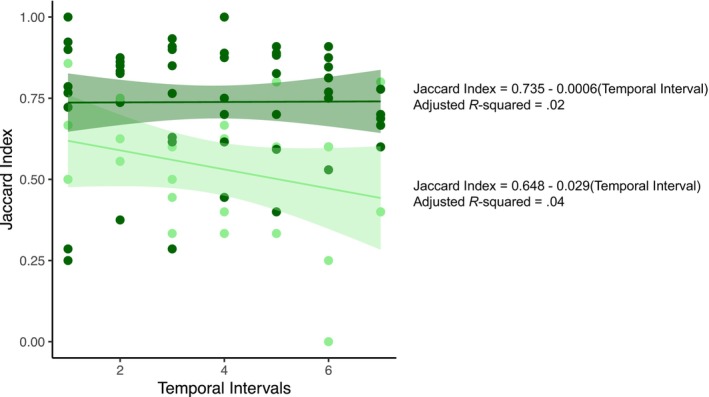
Temporal β‐diversity (Jaccard dissimilarity) of premontane (dark green) and basimontane (light green) sites. Temporal β‐diversity compares time *t* to time *t* + 1 for each site. Linear regressions are shown with 95% confidence intervals. Data represent Jaccard index for each site between consecutive samples, and adjusted *R* ^2^ values are low because trends are essentially flat through time, as indicated by nonsignificant slope coefficients for Temporal Interval (premontane *p* = .96, basimontane *p* = 17).

Premontane sites (<500 m) maintained greater temporal β‐diversity (Jaccard index) through time than basimontane sites (>500 m; Figure 5). As a reminder, temporal β‐diversity was calculated through time (i.e., *t* + 1 – *t*) for each site. Thus, lower elevation stream sites that are more vulnerable to human land use effects had more taxonomic heterogeneity than did the higher elevation sites.

Overall, for all of the diversity components evaluated herein, using altitudinal level as fixed effect and site and year as mixed effects was rather predictive, and no pre‐ and postconflict difference was yet apparent in results (i.e., results here represent conditions before further human land use effects). Fish diversity predictably decreased with altitude as a power law function, and fish communities were different in pre‐ and basimontane streams.

## 4 DISCUSSION

Our study is the first of its kind to evaluate fish community structure in the Colombian Andes‐Amazon transition zone. Here we had the opportunity to evaluate altitudinal gradients using altitude from two perspectives, as a continuous and a categorial variable. Results here provide a baseline for conservation of regional streams across premontane and basimontane levels because data represent the period when armed conflict ended but before potential development of formerly avoided lands (Calle‐Rendón et al., 2018).

Our results confirmed that altitudinal gradients in fish community assembly are important (van der Hammen & dos Santos, 1995) and that fish diversity is greater at lower altitudes, no matter how we estimated diversity (e.g., Jaramillo‐Villa et al., 2010; Lomolino et al., 2010; Lujan et al., 2013). Most of the times it is difficult to understand process causing patterns due to the many mechanisms that might be involved (Ricklefs, 2004). We provide evidence that deterministic processes are more important for freshwater fish diversity patterns along an altitudinal gradient; otherwise, patterns would not be consistent in space and time. Furthermore, fish diversity patterns are strongly associated with altitude in our study system, whether altitude is viewed as a categorical (basimontane and premontane) or continuous predictor. The strong altitude gradient and changes in associated stream conditions appear to be responsible for deterministic processes at local scales (Dyer et al., 2007; Schemske et al., 2009) that are stronger than regional scale mechanisms related to dispersal that may blur the strong gradient (Qian & Ricklefs, 2007; Ricklefs, 1987). Considering that effective mitigation of anthropogenic pressures on streams and fish assemblages should account for site‐specific conditions and at different scales (Newbold et al., 2015; Poiani et al., 2000), altitudinal gradients in the study area may be predictive for conservation and management. We note that the basimontane streams are important to conserve despite their relatively low diversity because fishes there are notably different from those downstream.

Changes in community composition through time are typically complex and depend on spatial and land use contexts (Allen et al., 2019; Hill et al., 2021). We expect that fish diversity might be most heavily affected at the premontane level over time due to human activities that will stronger and earlier at lower altitudes. Conservation practices (e.g., riparian buffer zones, runoff settling ponds) to maintain stream conditions can be implemented early in regional land changes to best conserve diversity.

Given species extinction rates due to habitat degeneration in ecosystems similar to those sampled here (Manjarrés‐hernández et al., 2021), this study is valuable as a baseline of barely impacted conditions as the conflict ended in Colombia and before encroaching development of formerly avoided lands (Calle‐Rendón et al., 2018). We expect future sampling will compare results to those reported here to document changes in fish assemblages due to land use changes. We also expect land use to be most changed at lower altitudes, where soils and slopes are more amenable to agriculture, roads, and housing. If so, then it is possible that fish assemblages will be degraded by increased land use to become simpler and later affected by upstream effects (Figure 1). If ongoing deforestation and encroaching anthropogenic land use in the hyper‐diverse Amazon basin is to be managed to minimize species losses, then some lands and streams must be preserved (Granado‐Lorencio et al., 2007; Mckinney & Lockwood, 1999; Rull, 2007). Results here suggest that land surrounding premontane streams should take priority to conserve the most species in areas that are most vulnerable. Upstream catchments in basimontane areas can also be preserved to maintain water quality flowing into premontane streams and conserve unique fishes in those upstream reaches. Future work in the study area should evaluate relative effects of changing land use in upper and lower catchments on fish diversity in the lower reaches.

So far, most emphasis on ecological studies on understanding diversity has been on spatial patterns of biological diversity rather than analyzing temporal patterns. Understanding temporal changes is essential to predict possible scenarios of the most diverse world's biota in natural ecosystems of the Amazon. Fish assemblages are affected by processes occurring at multiple scales, including those covered in our analyses (Jackson et al., 2001; Livingstone et al., 1982; Oberdoff et al., 2011; Tedesco et al., 2005). Future surveys could integrate data at scales obtained here with even greater spatial and temporal extents. We regard results here as a potential reference for future work in the same drainages, where future work may reveal effects of coming anthropogenic land use in the Amazon basin. Our data integrated spatial and temporal analysis to elucidate the consistent altitudinal gradient under relatively unimpacted conditions. By implication, similar conditions exist to be conserved in other headwaters that host the most diverse fish fauna in the world.

## AUTHOR CONTRIBUTIONS

**David G. Jenkins:** Conceptualization (lead); formal analysis (lead); methodology (lead); writing – review and editing (lead). **Astrid Acosta‐Santos:** Data curation (equal). **Edwin Agudelo Córdoba:** Resources (supporting). **Juan David Bogota‐Gregory:** Conceptualization (lead); data curation (lead); formal analysis (lead); investigation (lead); methodology (lead); project administration (lead); resources (lead); writing – original draft (lead).

## CONFLICT OF INTEREST STATEMENT

All authors declare that there are no competing interests.

## Supporting information


Figure S1

Appendix S1

Table S1

## Data Availability

Supporting data are available at https://doi.org/10.5281/zenodo.10424407.
