# Supplementary material for: Fish diversity of Colombian Andes‐Amazon streams at the end of conflict is a reference for conservation before increased land use
Source: Ecol Evol. 2024 Mar 13;14(3):e11046. doi: 10.1002/ece3.11046 (PMC10937820; doi:10.1002/ece3.11046)
Supplement: Supplementary file 1 — Figure S1 [file ECE3-14-e11046-s003.docx]

**Online Resource**

**Supplementary Figures**

**Fish diversity of post-conflict Colombian Andes-Amazon streams as a reference for conservation before increase land use**

Juan David Bogota-Gregory^1^*, David G. Jenkins^2^, Astrid Acosta-Santos^1^, Edwin Agudelo Córdoba^1^

*Corresponding author email: [juandbogota@gmail.com](mailto:juandbogota@gmail.com)


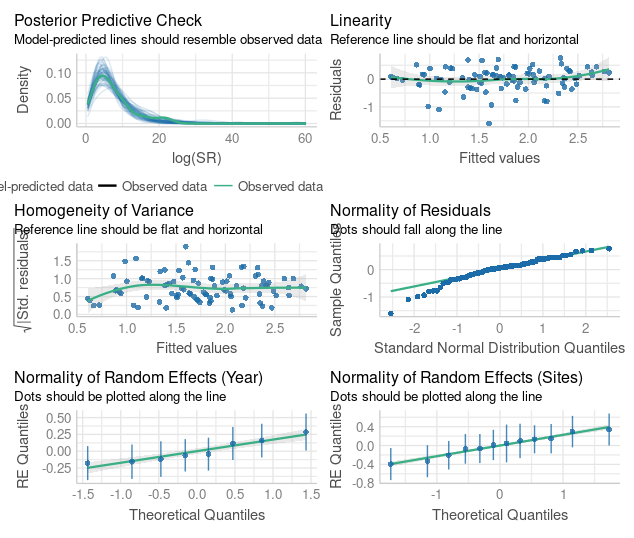


Figure S1. Model performance for species richness. Conditional R^2^ = 0.622. Marginal R^2^ = 0.421.

**
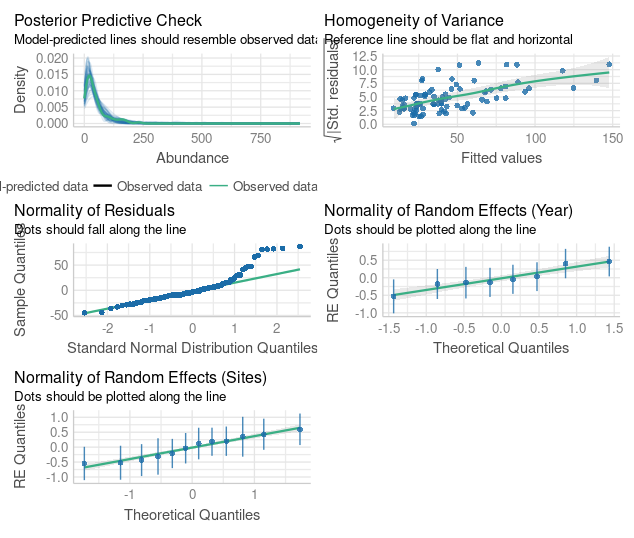
**

Figure S2. Model performance for species richness. Conditional R^2^ = 0.480. Marginal R^2^ = 0.131.

**
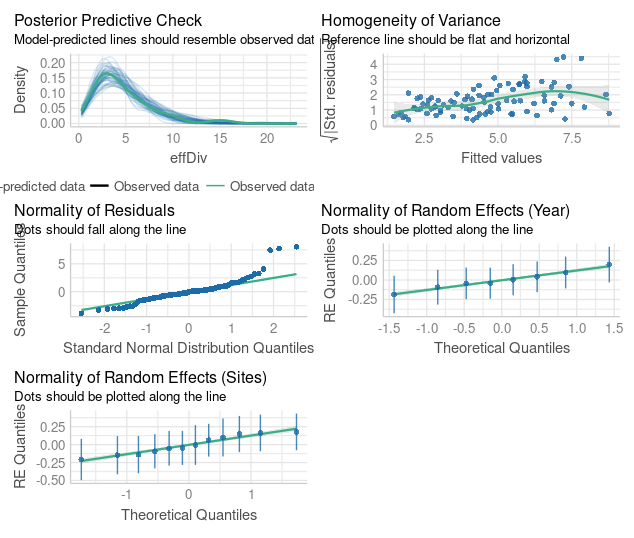
**

Figure S3. Model performance for species richness. Conditional R^2^ = 0.4567. Marginal R^2^ = 0.433.
