# Supplementary material for: Fish diversity of Colombian Andes‐Amazon streams at the end of conflict is a reference for conservation before increased land use
Source: Ecol Evol. 2024 Mar 13;14(3):e11046. doi: 10.1002/ece3.11046 (PMC10937820; doi:10.1002/ece3.11046)
Supplement: Supplementary file 3 — Table S1 [file ECE3-14-e11046-s002.docx]

**Online Resource**

**Supplementary Tables**

**Fish diversity of post-conflict Colombian Andes-Amazon streams as a reference for conservation before increase land use**

Juan David Bogota-Gregory^1^*, David G. Jenkins^2^, Astrid Acosta-Santos^1^, Edwin Agudelo Córdoba^1^

*Corresponding author email: [juandbogota@gmail.com](mailto:juandbogota@gmail.com)

Table S1. Species richness model selection results, based on the Akaike Information Criterion (corrected for small sample size; AICc). Model selection emphasized weight (i.e., probability that a model is most efficient among those listed), where selection was most clear-cut if δAICc of the next model was > 2.

| Predictors | AICc | δAICc | terms | weight |
| --- | --- | --- | --- | --- |
| Altitude + Random Effects (REs) | 146.3 | 0.0 | 5 | 0.749 |
| Altitude + conflict + REs | 148.5 | 2.2 | 6 | 0.250 |
| Null + REs | 158.4 | 12.1 | 4 | 0.002 |
| Conflict + REs | 160.5 | 14.2 | 5 | < 0.001 |
| Null | 203.8 | 57.5 | 2 | < 0.001 |

Table S2. Total abundance model selection results, based on the Akaike Information Criterion (corrected for small sample size; AICc). All information as in Table S2.

| Predictors | AICc | δAICc | terms | weight |
| --- | --- | --- | --- | --- |
| Altitude + Random Effects (REs) | 855.9 | 0.0 | 5 | 0.568 |
| Null + REs | 858.1 | 2.2 | 4 | 0.188 |
| Altitude + conflict + REs | 858.2 | 2.3 | 6 | 0.182 |
| Conflict + REs | 860.4 | 4.4 | 5 | 0.062 |
| Null | 884.5 | 28.5 | 2 | < 0.001 |

Table S3. Effective diversity model selection results, based on the Akaike Information Criterion (corrected for small sample size; AICc). All information as in Table S2.

| Predictors | AICc | δAICc | terms | weight |
| --- | --- | --- | --- | --- |
| Altitude + Random Effects (REs) | 371.6 | 0.0 | 5 | 0.620 |
| Altitude + conflict + REs | 372.6 | 1.0 | 6 | 0.380 |
| Null + REs | 386.2 | 14.7 | 4 | < 0.001 |
| Conflict + REs | 387.2 | 15.7 | 5 | < 0.001 |
| Null | 424.3 | 52.8 | 2 | < 0.001 |
